# Chloroquine potentiates the anti-cancer effect of 5-fluorouracil on colon cancer cells

**DOI:** 10.1186/1471-2407-10-370

**Published:** 2010-07-15

**Authors:** Kazuhito Sasaki, Nelson H Tsuno, Eiji Sunami, Giichiro Tsurita, Kazushige Kawai, Yurai Okaji, Takeshi Nishikawa, Yasutaka Shuno, Kumiko Hongo, Masaya Hiyoshi, Manabu Kaneko, Joji Kitayama, Koki Takahashi, Hirokazu Nagawa

**Affiliations:** 1 Department of Surgical Oncology, Faculty of Medical Sciences, the University of Tokyo, Tokyo 113-8655, Japan; 2 Department of Transfusion Medicine, Faculty of Medical Sciences, the University of Tokyo, Tokyo 113-8655, Japan

## Abstract

**Background:**

Chloroquine (CQ), the worldwide used anti-malarial drug, has recently being focused as a potential anti-cancer agent as well as a chemosensitizer when used in combination with anti-cancer drugs. It has been shown to inhibit cell growth and/or to induce cell death in various types of cancer. 5-Fluorouracil (5-FU) is the chemotherapeutic agent of first choice in colorectal cancer, but in most cases, resistance to 5-FU develops through various mechanisms. Here, we focused on the combination of CQ as a mechanism to potentiate the inhibitory effect of 5-FU on human colon cancer cells.

**Methods:**

HT-29 cells were treated with CQ and/or 5-FU, and their proliferative ability, apoptosis and autophagy induction effects, and the affection of the cell cycle were evaluated. The proliferative ability of HT-29 was analyzed by the MTS assay. Apoptosis was quantified by flow-cytometry after double-staining of the cells with AnnexinV/PI. The cell cycle was evaluated by flow-cytometry after staining of cells with PI. Autophagy was quantified by flow-cytometry and Western blot analysis. Finally, to evaluate the fate of the cells treated with CQ and/or 5-FU, the colony formation assay was performed.

**Results:**

5-FU inhibited the proliferative activity of HT-29 cells, which was mostly dependent on the arrest of the cells to the G0/G1-phase but also partially on apoptosis induction, and the effect was potentiated by CQ pre-treatment. The potentiation of the inhibitory effect of 5-FU by CQ was dependent on the increase of p21^Cip1 ^and p27^Kip1 ^and the decrease of CDK2. Since CQ is reported to inhibit autophagy, the catabolic process necessary for cell survival under conditions of cell starvation or stress, which is induced by cancer cells as a protective mechanism against chemotherapeutic agents, we also analyzed the induction of autophagy in HT-29. HT-29 induced autophagy in response to 5-FU, and CQ inhibited this induction, a possible mechanism of the potentiation of the anti-cancer effect of 5-FU.

**Conclusion:**

Our findings suggest that the combination therapy with CQ should be a novel therapeutic modality to improve efficacy of 5-FU-based chemotherapy, possibly by inhibiting autophagy-dependent resistance to chemotherapy.

## Background

Colorectal cancer is a leading cause of cancer-related death in developed countries [1]. Surgical resection, followed by adjuvant chemo or radiotherapy, remains the only established curative treatment for colon cancer, but this treatment is often unsatisfactory in case the patient present with the disease in an advanced stage or metastasis develops after attempted curative resection.

5-Fluorouracil (5-FU) is the chemotherapeutic agent of first choice in the treatment of patients with colorectal cancer. Intracellular metabolites of 5-FU can exert cytotoxic effects via inhibition of thymidylate synthetase, or through incorporation into RNA and DNA, events that ultimately activate apoptosis [2]. The dose increment of systemic administration of 5-FU would generate unacceptable levels of toxicity to normal cells, especially of bone marrow and gastrointestinal tract, resulting in severe adverse effects. Therefore, many attempts have been made to enhance its therapeutic effectiveness, simultaneously reducing its toxicity. However, presently, the ideal chemotherapeutic agent or the best combination of agents, which should cause strong cytotoxicity against cancer cells, with minimal effect on normal cells, has not yet been developed.

Chloroquine diphosphate (CQ), a worldwide used anti-malarial drug, has recently been focused due to its potential biological effects on cancer cells, such as the inhibition of cell growth and/or induction of cell death in human lung cancer A549 cells, glioma cells, human breast cancer cells, and mouse colon cancer CT26 cells, resulting in anti-cancer effects [3-7]. And CQ has been shown to potentiate the inhibitory effect of GX15-070, a novel proapoptotic anti-tumor agent that inhibit anti-apoptotic Bcl-2 proteins, on the growth of esophageal cancer cells [8]. Additionally, CQ has been shown to inhibit the antitumor effect of tephrosin, a natural rotenoid that induces internalization of EGFR and ErbB2, with consequent degradation of these receptors, on colon cancer cells. This effect was dependent on the blocking of the degradation of the receptors internalized into vesicles [9].

In addition to its anti-cancer effects, CQ is a well-known lysosomotropic agent [10,11]. The lysosomotropic properties of CQ are probably responsible for many of the biological effects of this drug. Recently, accumulating lines of evidence suggest that, through its lysosomotrophic effect, CQ can effectively sensitize cancer cells to the cell-killing effects of ionizing radiation and chemotherapeutic agents and, therefore, its use as an effective sensitizer for the improvement of the effect of conventional cancer therapies is a promising new therapeutic strategy [12]. Hu et al have demonstrated the effectiveness of CQ as a cancer-specific chemosensitizer in combination with Akt inhibitors [13].

The cancer-specific chemosensitizer effect of CQ may be partly dependent on its ability to inhibit autophagy [10-12]. It was reported to specifically inhibit autophagy in a mechanism distinct from other autophagy inhibitors, such as 3-methyladenine (3-MA). Whereas 3-MA inhibits autophagy in its early phase, consequently resulting in inhibition of the formation of acidic vesicular organelles (AVOs), which consist predominantly of autophagosomes and autolysosomes, CQ inhibits autophagy in its late phase, i.e., after AVOs have been formed in the cytoplasm of the cells and, therefore, CQ-treated cells show a typical feature of AVOs accumulation in the cytoplasm [11]. Inhibition of autophagy, therefore, is a promising new strategy to improve cancer treatment. In a previous study, inhibition of autophagy with 3-MA in colon cancer cell lines exposed to 5-FU resulted in reduced cell survival [14]. And 3-MA was shown to enhance 5-FU-induced apoptosis in colon cancer cells, in vitro and in vivo suggestive that autophagy might play a role as a self-defense mechanism in 5-FU-treated colon cancer cells [15,16]. We have also demonstrated that inhibition of autophagy in colon cancer cells with 3-MA potentiated the proapoptotic effect of sulforaphane, a kind of isothiocyanate, with strong pro-apoptotic effects against cancer cells as well as endothelial cells [17]. Taking these facts, CQ, through its lysosomotropic properties as well as the autophagy inhibition ability, may be a promising agent to be used in combination with chemotherapeutic agents for the improvement of clinical results.

Since its introduction into clinical practice in 1947 for the prophylaxis treatment of malaria (Plasmodium vivax, ovale and malariae), CQ still remains the drug of choice for malaria chemotherapy, because of its high effectiveness and well tolerance by humans [12]. In addition, CQ is widely used as an anti-inflammatory drug for the treatment of rheumatoid arthritis, lupus erythematosus and amoebic hepatitis. Therefore, the great advantage of CQ over other inhibitors of autophagy, including 3-MA, is the feasibility of its introduction to the clinical settings of cancer therapy without need of animal or phase-one studies. Since CQ and 3-MA inhibit different steps of the autophagic process, there is need to confirm the effectiveness of CQ in potentiating the anti-cancer effect of 5-FU against colon cancer cells, in a similar mechanism as 3-MA.

In the present study, therefore, we aimed to investigate the effect of the combination therapy of 5-FU with CQ on human colorectal cancer cells, namely HT-29. Cells were treated with CQ or/and 5-FU, and their proliferative ability, induction of apoptosis, and the changes of cell cycle were evaluated. In addition, the ability of HT-29 to induce autophagy in response to 5-FU, and the affection of autophagy by CQ were analyzed. Our results demonstrated that treatment with CQ, at a relatively low concentration for a short period of time, although without a direct inhibitory effect on HT-29 cell proliferation and cell growth, potentiated the inhibitory effect of 5-FU on the proliferative activity, which was dependent on the cell cycle arrest in the G0/G1 phase.

## Methods

### Cells and Reagents

The human colorectal cancer cell line, HT-29, obtained from the Japanese Cancer Research Resource Bank, was used. Cells were cultured in RPMI-1640 medium supplemented with 5% fetal calf serum, 1% antibiotics/antimycotic (complete medium) and incubated in a 5% CO_2 _incubator at 37°C. 5-fluorouracil (FU), Chloroquine diphosphate (CQ), bovine serum albumin, and RPMI-1640 medium were purchased from Sigma-Aldrich (St Louis, MO, USA). Fetal calf sera, and antibiotics/antimycotics were from Gibco BRL (Grand Island, NY, USA). Calcium and magnesium-free phosphate-buffered saline (PBS (-)) was from Wako Pure Chemical Industries, Ltd (Osaka, Japan). Acridine orange and trypan blue were from Sigma-Aldrich Japan (Tokyo, Japan).

### 5-fluorouracil and Chloroquine treatment

Prior to each treatment, HT-29 cells were grown until they reached about 30-50% subconfluence. Then, to test the independent effects of 5-FU and CQ on HT-29, the cells were treated with 5-FU at 0.01, 0.1, 1, 10, 100, or 1000 μM for 24, 48, and 72 h, or with CQ at 0.1, 1, 10, 100, or 1000 μM CQ for 12, and 24 h, in an attempt to determine the dose of CQ that effectively inhibited autophagy without affecting the proliferative activity of HT-29. Additionally, the dose of 5-FU and the treatment time necessary for the 50% inhibition (CI50) of the proliferative activity of HT-29 was determined.

To assess the effects of the co-treatment with 5-FU and CQ, the cells were pre-treated with CQ at the dose of 80 μM for 12 h, and after washing with PBS, cells were treated with the determined doses of 5-FU for another 48 h. The dose of CQ was set at 80 μM because it was the dose that mostly induced AVOs formation, the marker of autophagy, with minimal inhibitory effect on HT-29 cells.

In the subsequent experiments, cells were pre-treated with CQ (80 μM) for 12 h, followed by 5-FU (5 μM) for another 24-48 h.

### Cell proliferation assay and Cell growth assessment

In the preliminary experiment, cells were treated with either CQ or 5-FU at the various concentrations for the different periods of time, and in the subsequent experiments, by CQ plus 5-FU, as above described. The MTS assay was used for the assessment of cell proliferation, and cell growth was assessed by counting live cells after staining with trypan blue. Cell Titer 96TM aqueous nonradioactive cell proliferation assay (Promega, Madison, WI, USA) was used according to the manufacturer's recommendation. The assay consisted of tetrazolium compound (inner salt, MTS) and an electron coupling reagent, phenazine methosulfate (PMS). MTS is converted by the dehydrogenase enzymes into a formazan, which is found in metabolically active cells and is soluble in tissue culture medium. The absorbance of the formazan at 490 nm was measured directly in a 96-well plate using a microtiter plate reader (Beckton Dickinson). Dehydrogenase enzymes found in metabolically active cells accomplish the conversion of MTS into the aqueous-soluble formazan. The quantity of formazan product, and thus the amount of 490 nm absorbance, is directly proportional to the number of living cells in the culture. The treated and untreated control cells were cultured in 96-well flat-bottomed plates at a density of 5 × 10^3 ^cells per well in 100 μl complete medium, at 37°C in an atmosphere of 5% CO_2_. Then, 20 μl of the MTS solution was added to each well and incubated at 37°C for 3-4 hours. All experiments were performed in triplicate wells, and the proliferation of HT-29 cells was calculated as the ratio of each experimental condition to the control one (untreated cells).

### Detection of Acidic vesicular organelles (AVOs)

For the analysis of autophagy induction, acidic vesicular organelles (AVOs), which consist predominantly of autophagosomes, and autolysosomes, were quantified by flow cytometry after staining of cells with acridine orange (AO). AO is a fluorescent weak base that accumulates in acidic spaces and fluoresce bright red. The intensity of the red fluorescence is proportional to the degree of acidity. Thus, AO fluorescence intensity, which represents AVOs formation, can be quantified by flow-cytometry. HT-29 cells were prepared and treated as described, and the cells were collected in FACS tubes (BD Biosciences Discovery Labware, MA, USA) and resuspended in PBS (-). The cell suspension was stained with AO (5 μg/ml) for 15 min at room temperature. The cells were washed twice with PBS (-), resuspended in PBS and analyzed on a flow-cytometer using the CellQuest software, and examined under a fluorescence microscope at 40 × objective lens magnification. The experiment was performed three times with essentially the same results.

### Immunocytochemistry for LC3 localization

HT-29 cells were prepared and treated as described, washed with PBS (-) and fixed with 4% paraformaldehyde at 4°C overnight. Subsequently, the cells were permeabilized with 0.1% Tween-20 for 15 minutes at room temperature, and blocked with 0.2% bovine serum albumin (BSA) for 1 hour at room temperature. The cells were treated with anti-LC3 antibody (MBL, Nagoya, Japan) for 1 hour at room temperature. Cells were then washed with BSA buffer and incubated with 2 μg/mL Alexa Fluor 488-conjugated goat anti-rabbit antibody (Eugene, OR, USA) for 1 hour at room temperature and examined under a fluorescence microscope at 40 × objective lens magnification.

### Detection of apoptosis by flow-cytometry

HT-29 cells were prepared and treated as described, and then stained with fluorescein isothiocyanate (FITC)-conjugated annexin V and propidium iodide (PI) for 5 minutes at room temperature according to manufacturer's instructions (AnnexinV: FITC Apoptosis Detection Kit, BD Pharmingen, CA, USA). The population of annexin V^-^PI^- ^viable cells and annexin V^+ ^apoptotic cells was evaluated by flow-cytometry, and the later were considered apoptotic cells. Data were collected in a FACS Calibur (Becton-Dickinson, Mountain View, Calif) and analyzed by using the Cell Quest software (Becton-Dickinson). The experiment was performed three times, and the ratio of apoptotic cells was expressed as mean ± SD.

### Analysis of the cell cycle by flow-cytometry

HT-29 cells were prepared and treated as described, and then the percentage of cells in each phase of the cell cycle was analyzed by flow-cytometry, using the Cycle TEST PLUS DNA Reagent Kit (BD Pharmingen, San Jose, CA, USA), which is based on the measurement of the DNA content of nuclei labeled with propidium iodide (PI), according to manufacturer's instructions. Treated cells were trypsynized (250 μL of trypsin buffer) for 10 min at room temperature, and then trypsin inhibitor (200 μL) and RNase buffer were added and allowed to react for 10 min at room temperature. Finally, propidium iodide stain solution (200 μL) was added and incubated for 10 min in the dark, on ice. Samples were immediately analyzed in the flow-cytometer (Becton-Dickinson, Mountain View, Calif), and the obtained results analyzed by the Cell Quest software (Becton-Dickinson). The experiment was performed three times, and the ratio of cells in the G0/G1, intra-S, and G2/M phases were expressed as mean ± SD.

### Colony forming assay

HT-29 cells, suspended in fresh culture medium, were plated 200 cells/well onto 24-well microtiter plates. After 24 hours of culture, to allow cells to attach, cells were treated with CQ and/or 5-FU, respectively, at 80 or 5 μM for 12 or 48 hours. Then, cells were washed with PBS (-), and fed with fresh culture medium, without5-FU, and allowed to grow for 11 days in normal culture conditions (37°C, 5%CO_2_). During the 11 days of culture, the colonies that contained 50 or more cells were counted under light microscope. For each experimental condition, colonies from 3 different wells were counted. The proliferative ability of HT-29 cells was calculated as the ratio of the experimental condition to the control one at day 5 (untreated cells).

### Western Blot Analysis

Cells were treated as above described, and. after 3 times washing with PBS(-), cells were harvested by scraping the culture dishes with a cell scraper and subsequently lysed with 0.5 ml of Tris-saline (50 mM Tris-HCl (pH 7.6), 150 mM sodium chloride) containing various protease inhibitors (1 mM EGTA, 0.1 mM diisopropyl fluorophosphates, 0.5 mM phenylmethyl-sulphonyl fluoride, 1 mg/ml Na-ρ-tosyl-L-lysine chloromethyl ketone, 1 mg/ml anti-pain, 0.1 mg/ml pepstain, 1 mg/ml of leupeptin) and 1% Triton-X for 1 hour in cold room. After centrifuged at 15,000 rpm for 5 minutes, the clear supernatant was collected and used as the cell protein extract. Protein concentration was determined using the BCA Protein Assay Kit (Pierce Biomedical Co, Rockford, IL). Cell lysates were separated on a 15% Ready Gel J SDS-PAGE (Bio-Rad, Hercules, CA, USA) for Hybond ECL nitrocellulose membrane blotting (Amersham Pharmacia Biotech, Buckinghamshire, England). The blotted membranes were blocked with 5% skim milk for 1 hour and were incubated with each primary antibody overnight at 4°C. The following primary antibodies were used: anti-Cdk2, -Cdk4, -Cdk6, -β-actin were purchased from Santa-Cruz Biotechnology (Santa Cruz, CA, USA). LC3, cyclinD1, cyclin E were from Medical Biological Laboratories CO (Nagoya, Japan). p27^Kip1^, p21^Cip1 ^were purchased from Cell Signaling Technology (Beverley, MA, USA). Following the primary antibody incubation, the blots were washed and incubated with 1:10000 dilution of biotinylated anti-rabbit IgG (Vector Laboratories, Inc, Burlingame, CA, USA) or biotinylated anti-mouse IgG (Vector Laboratories, Inc, Burlingame, CA, USA), as appropriate. The immunoreactive bands were visualized by enhanced chemiluminescence using the ECL detection system (Amersham Pharmacia Biotech, Buckinghamshire, England). The density of each band was measured using Image J software (open source Image J software available at http://rsb.info.nih.gov/ij/).

### Statistical analysis

All of experiments were repeated at least three times. Statistical significance of the differences was evaluated by the unpaired, 2-tailed Student's t-test, and an association was considered significant when the exact significant level of the test was P < 0.05.

## Results

### Effect of 5-FU and CQ on the proliferative activity of HT-29 cells

**Figure 1:**
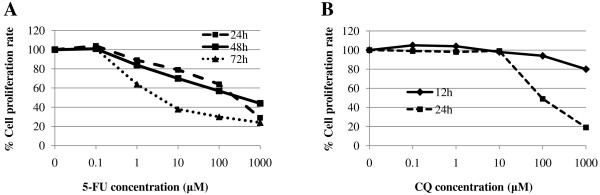
**Effect of 5-FU and CQ on the proliferative activity of HT-29 cells**. (A) The proliferative activity of 5-FU-treated HT-29 cells for 24, 48, 72 hours assessed by the MTS assay. The y-axis represents the proliferation rate, calculated as the ratio to control untreated cells. The 5-FU-induced inhibitory effect of HT-29 cells was time and dose-dependently increased. (B) The proliferative activity of CQ-treated HT-29 cells for 12 and 24 hours assessed by the MTS assay. The y-axis represents the proliferation rate, calculated as the ratio to control untreated cells. CQ treatment at 1000 μM for 12 h or 100 μM and higher doses for 24 h resulted in significant inhibition of the proliferative activity of HT-29 cells, but not at lower doses. Values were given as mean.

5-FU, at doses lower than 0.01 μM, had almost no inhibitory effect on HT-29 cells, after 24, 48 or 72 hr treatment. At the concentrations over 0.1 μM, 5-FU inhibited the proliferation of HT-29 cells in time- and dose-dependent manner (Fig. 1A). On the other hand, CQ at doses lower than 100 μM in a 12 h-treatment, or at 10 μM in a 24 h-treatment, had minimal inhibitory effect on HT-29 cells (Fig. 1B). Based on these results, the dose of 80 μM of CQ in a 12 h-treatment, which had no inhibitory effect on HT-29 cells, and that of 5 μM of 5-FU for 48-72 h, which had around 50% inhibitory effect, were chosen for the further experiments.

### Effect of CQ on 5-FU-induced cell proliferation and growth inhibition

**Figure 2:**
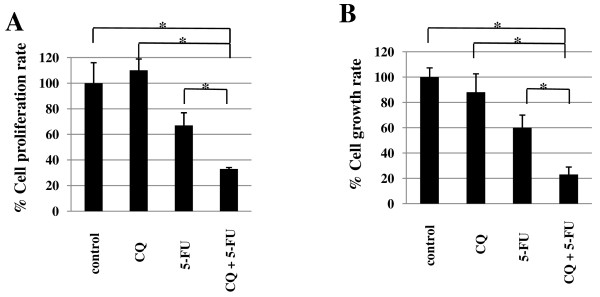
**Effect of CQ in 5-FU-induced cell proliferation and growth inhibition**. The effect of CQ-pretreatment on the inhibitory effect of 5-FU on the proliferative activity/cell growth of HT-29 cells was investigated by MTS assay/trypan blue exclusion staining. The y-axis represents the proliferation/growth rate, calculated as the ratio to control untreated cells. Values were given as mean ± SD. (A) 5-FU at 5 μM is reported to cause minimal adverse health effects to human beings. Pre-treatment of cells with CQ at 80 μM for 12 hours prior to exposure to 5-FU at 5 μM for 48 hours, resulted in potentiation of the inhibitory effect (33% vs. 67% inhibition for 5-FU alone and CQ + 5-FU, respectively). (*, *p *< 0.05 vs. control) (B) When cells were treated with CQ at 80 μM for 12 hours prior to exposure to 5-FU at 5 μM for 48 hours, the growth rate reduction was potentiated (60% vs 23% live cells for 5-FU alone and CQ + 5FU, respectively). (*, *p *< 0.05 vs. control)

Treatment with 5-FU at 5 μM for 48 hours resulted in inhibition of the proliferative activity of HT-29 to approximately 67% of untreated control cells. Pre-treatment of cells with CQ at 80 μM for 12 h prior to exposure to 5-FU enhanced the inhibitory effect, the proliferative activity decreasing to approximately 33% of the control (Fig. 2A). Then, cell viability was assessed by the trypan blue exclusion staining. Similar to the results of the MTS assay, pre-treatment of cells with CQ resulted in potentiation of the growth inhibition rate (60% vs 23% live cells for 5-FU alone and CQ+5FU, respectively) (Fig. 2B).

### CQ increased the formation of Acidic vesicular organelles (AVOs), and the expression of LC3-II in 5-FU-treated HT-29 cells

**Figure 3:**
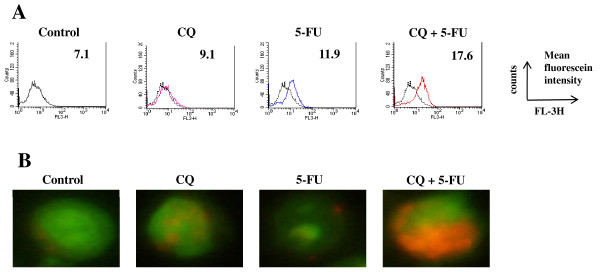
**CQ increased the formation of Acidic vesicular organelles (AVOs)**. (A) The formation of AVOs was quantified by flow-cytometry after acridine orange staining in HT-29 cells treated with 5-FU for 48 h after 12-h pretreatment with CQ. Treatment of HT-29 cells with 5-FU alone resulted in increased AVOs formation, but the effect was potentiated by CQ-pretreatment. The results are expressed as the mean fluorescein intensity of acridine orange stained cells. (B) The similar results were obtained with the fluorescence microscopic examination, confirming the induction of autophagy. HT-29 cells treated with CQ and 5-FU displayed great number of red fluorescence vesicles in the cytoplasm, which represent AVOs. Because CQ inhibits the late step of autophagy, the accumulation, and not inhibition, of AVOs was induced by the treatment with CQ.

**Figure 4:**
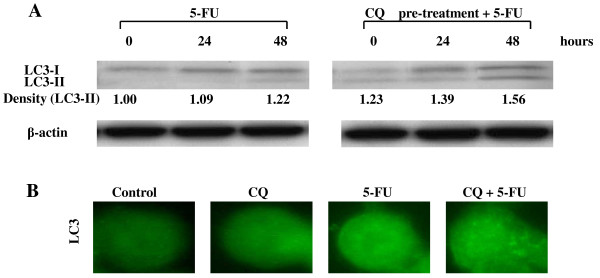
**CQ potentiates the accumulation of LC3-II induced by 5-FU**. (A) The expression of LC3-II was quantified by Western blot in HT-29 cells treated with 5-FU alone (left) for 48 h or after 12-h pretreatment with CQ (right). Treatment of HT-29 cells with 5-FU alone resulted in increased accumulation of LC3-II time-dependently, and the effect was potentiated by CQ-pretreatment. The expression levels of LC3-II are expressed as the density measured by the Image J software, standardized by the density of β-actin. (B) The intracellular localization of LC3 was analyzed by immunofluorescence microscopy, by staining the cells with fluorescent antibodies to LC3. Control (untreated) and CQ-treatment cells exhibited a weak and diffuse cytoplasmic staining with LC3-associated green fluorescence, whereas those treated with 5-FU and CQ pre-treatment + 5-FU exhibited an evident punctuate green fluorescence pattern of LC3, which is a typical feature of LC3 distribution within autophagosomes (LC3-II).

Treatment of HT-29 cells with 5-FU at 5 μM for 48 hours resulted in increased AVOs formation, compared with untreated cells. In addition, the AVOs formation was also increased by the treatment with the combination of CQ and 5-FU, compared with cells treated with 5-FU alone (Fig. 3A). Similar results were obtained with the fluorescence microscopic examination, confirming the induction of autophagy. HT-29 cells treated with CQ and 5-FU displayed great number of red fluorescence vesicles in the cytoplasm, which represent AVOs, compared with cells treated with 5-FU alone (Fig. 3B). Then, to confirm autophagy induction by 5-FU, and the inhibition by CQ, the expression of LC3-II was investigated in HT-29 cells treated with 5-FU at 5 μM for 24, and 48 hours, by Western blot. The treatment of HT-29 cells with 5-FU alone resulted in increased accumulation of LC3-II, which was observed time-dependently, and the effect was potentiated by CQ-pretreatment (Fig. 4A). The intracellular localization of LC3 by immunofluorescence microscopy, after staining of the cells with fluorescent antibodies to LC3, revealed an evident punctuate green fluorescence pattern of LC3 in cells treated with 5-FU alone or the combination of CQ+5-FU, the typical feature of LC3 distribution within autophagosomes (LC3-II). On the other hand, control untreated cells and cells treated with CQ alone exhibited only a weak and diffuse cytoplasmic staining with LC3-associated green fluorescence (Fig 4B). Because CQ inhibits the late step of autophagy, the accumulation, but not inhibition, of AVOs and LC3-II were induced.

### CQ potentiated the arrest of HT-29 cells to the G0/G1 phase of the cell cycle, and the apoptosis induced by 5-FU

**Figure 5:**
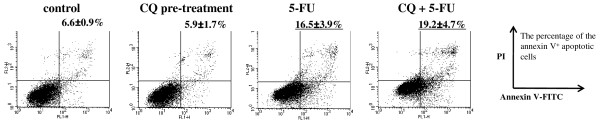
**CQ potentiates apoptosis of HT-29 cells induced by 5-FU**. The population of annexin V^+ ^apoptotic cells were evaluated by FCM using annexin V-FITC/PI staining in HT-29 cells after CQ-pretreatment at 80 μM for 12 hours, followed by 5-FU at 5 μM for 48 hours. The percentage of annexin V^+ ^apoptotic cells increased by pretreatment with CQ followed by 5-FU (16.5% vs 19.2% for 5-FU alone and CQ+5-FU, respectively). Values were given as mean ± SD.

The percentage of apoptotic cells (annexin V^+^) were slightly increased by pretreatment with CQ followed by 5-FU (16.5% vs 19.2%; 5-FU alone vs CQ and 5-FU) (Fig. 5).

**Figure 6:**
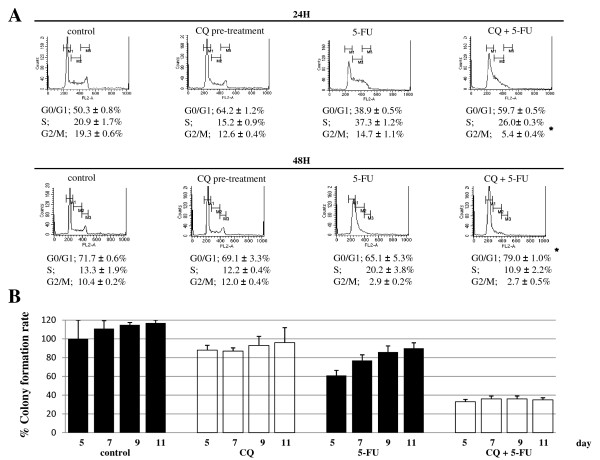
**CQ potentiates the G0/G1 arrest induced by 5-FU**. (A) The analysis of changes in cell cycle was quantified by flow-cytometry after PI staining in HT-29 cells treated without or with 5-FU for 24, 48 h after 12-h pretreatment without or with CQ. Values were given as mean ± SD. Treatment of HT-29 cells with 5-FU alone resulted in increased intra-S arrest, but the G1 arrest was potentiated by CQ-pretreatment. Furthermore, G2/M progression of HT-29 cells was blocked by the treatment with 5-FU alone, and it was potentiated by the 12-h pretreatment of CQ (*, *p *< 0.05 vs. control). (B) The colony formation rate was quantified in HT-29 cells treated without or with 5-FU for 48 h after 12-h pretreatment without or with CQ. Values were given as mean ± SD. Treatment of cells with 5-FU alone resulted in a significant delay in the colony-forming ability, but at day 11 of culture, approximately 90% of the cells have formed colonies, suggestive that these cells were in a dormant state. Pre-treatment of cells with CQ prior to exposure to 5-FU resulted in potentiation of the inhibitory effect on the colony forming ability, which was reduced to approximately 35% of control untreated cells at day 11. CQ alone partially inhibited the colony forming ability of HT-29, but it was almost completely recovered by day 11.

Treatment of HT-29 cells with 5-FU alone for 48 hours resulted in increased intra-S cell cycle arrest, as well as the arrest of the cells to the G0/G1 phase, which were dose-dependent. Pre-treatment of HT-29 cells with CQ resulted in strong arrest of cells to the G0/G1 phase of the cell cycle, compared with cells treated with 5-FU alone (percentage of cells in G0/G1-phase: 65.1% vs 79.0% for 5-FU alone vs CQ and 5-FU, respectively). Furthermore, treatment with 5-FU alone for 24 hours resulted in the inhibition of G2/M progression of HT-29 cells, which was also potentiated by the pretreatment with CQ (14.7% vs 5.4%; 5-FU alone vs CQ and 5-FU, respectively) (Fig. 6A). The colony formation was performed to confirm the viability of the cells after the treatment with 5-FU and/or CQ. Cells were pre-treated without or with CQ for 12-h followed by 5-FU treatment for 48-h, and then allowed to grow in complete medium without CQ or 5-FU for 11 days. CQ treatment alone caused a slight inhibition of the colony-forming ability of HT-29 cells. Treatment of cells with 5-FU alone resulted in a significant delay of the colony-forming ability, but at day 11 of culture, approximately 90% of the cells have formed colonies, suggestive that these cells were in a dormant state. Furthermore, pre-treatment of cells with CQ at 80 μM for 12 h prior to exposure to 5-FU resulted in potentiation of the inhibitory effect on the colony-forming ability, which was reduced to approximately 35% of control untreated cells at day 11 of culture (Fig. 6B).

### The G0/G1 arrest of HT-29 cells induced by 5-FU and potentiated by CQ was dependent on the increase of p21^Cip1^and p27^Kip1^, and decrease of CDK2

**Figure 7:**
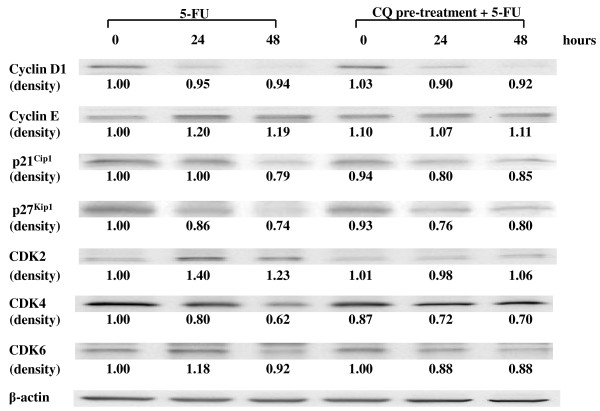
**Changes in the expression of cyclins, CDKs, CDKs inhibitors**. Changes in the expression of cyclins, CDKs, CDKs inhibitors were quantified by Western blot in HT-29 cells treated with 5-FU for 48 h after 12-h pretreatment with CQ. Treatment of HT-29 cells with 5-FU alone resulted in decreased expression of cyclin D1, p21^Cip1 ^and p27^Kip1^, time-dependently. In addition, 5-FU treatment resulted in accumulation of CDK2. Pre-treatment of cells with CQ resulted in inhibition of 5-FU-induced down-regulation of p21^Cip1 ^and p27^Kip1 ^expressions and, on the other hand, decreased the expression of CDK2. The expressions of cyclin E, CDK4 and CDK6 were not affected by 5-FU and/or CQ treatments. The expression levels of cyclins, CDKs, and CDKs inhibitors are expressed as the density measured by the Image J software, standardized by the density of β-actin.

Treatment of HT-29 cells with 5-FU alone resulted in decreased expression of p21^Cip1 ^and p27^Kip1^, and simultaneously in increased expression of CDK2, as detected by Western blot. Pre-treatment of cells with CQ inhibited the down-regulation of p21^Cip1 ^and p27^Kip1 ^expression induced by 5-FU and, on the other hand, decreased the expression of CDK2 (Fig. 7). The expression of cyclin D1 was down-regulated by the treatment with 5-FU, but not affected by the pre-treatment with CQ.

## Discussion

In the present study, we demonstrated the potentiation of the inhibitory effect of 5-FU on human colon cancer cells by CQ, the well-known lysosomotropic agent and the inhibitor of autophagy. Since some studies have suggested the direct cytotoxic effects of CQ on colon cancer cells, by inhibiting cell proliferation and inducing apoptosis [5], in the present study, preliminarily, we determined the dose of CQ necessary to inhibit autophagy without affecting cell viability. And the treatment of HT-29 cells with the combination of CQ plus 5-FU resulted in potentiation of the inhibitory effect on the proliferative activity, which was dependent on the enhancement of the cell cycle arrest to the G0/G1 phase, and also an increase in the percentage of apoptotic cells.

Interestingly, in HT-29 cells treated with 5-FU (5 μM) for 48 hours, the increased formation of AVOs and increased expression of LC3-II, compared with control untreated cells, were observed. These are the pathognomonic features of autophagy inhibition by CQ. Differing from the other inhibitors of autophagy, such as 3-MA, which inhibit autophagy in its early phase, consequently resulting in inhibition of AVOs formation, CQ inhibits autophagy in the late phase, when autophagosomes have already been formed, consequently resulting in accumulation of AVOs, as previously shown by others [10,18,19]. And the expression of LC3-II changes in parallel with AVOs. Therefore, we hypothesized that HT-29 cells induced autophagy as the defense mechanism against 5-FU, and the inhibition of autophagy by CQ could be responsible for the potentiation of the anti-cancer effect of 5-FU. Then, the colony formation assay was conducted to evaluate the behavior of the cells treated with CQ and/or 5-FU. Interestingly, the colony-forming ability of cells treated with CQ alone was partially inhibited, suggestive that autophagy is a mechanism necessary for cell survival under normal conditions. And more than 95% of the cells had formed colonies at day 11 of culture, confirming that CQ itself has minimal effect on colon cancer cells. On the other hand, 5-FU alone significantly inhibited the colony-forming ability of HT-29. However, at day 11 of culture, about 90% of the cells had formed colonies, which suggests that the cells were in a quiescent state, probably dependent on the induction of autophagy and cell cycle arrest to G0/G1-S-phase. The combination of CQ and 5-FU resulted in a more important inhibition of the colony-forming ability, which was not restored after 11 days in culture. Thus, we concluded that the autophagic process might be a protective event against the 5-FU-induced cell cycle arrest of cells to the G0/G1 phase, and that CQ, by inhibiting the autophagic process, enhanced the inhibitory effect of 5-FU on HT-29 cells.

In a recent report by Li J et al [15], 3-MA, another specific inhibitor of autophagy, was shown to enhance the effect of 5-FU-induced apoptosis in HT-29 cells. The basic difference between their report and ours is the inhibitor of autophagy used and the dose of 5-FU tested. CQ, similar to 3-MA, could efficiently inhibit autophagy in colon cancer cells, and inhibition of autophagy by CQ potentiated the anti-cancer effect of 5-FU, similar to 3-MA in this previous study. In the present study, however, we tested 5-FU at a dose lower than that in their study, which is a dose quite close to the physiologically achievable in human plasma [20]. And as a consequence, whereas they observed strong apoptosis induction by 5-FU, we found significant inhibition of the cell cycle to the G0/G1 phase. The relationship between autophagy and cell cycle is yet poorly known. It has been reported that rapamycin, an inducer of apoptosis and autophagy, leads cells to be arrested to the G0 phase [21], and that, in nutrient-deprived cells, autophagic vacuoles do not accumulate during the meta- and anaphases of mitosis [22]. In addition, it was reported that autophagy is closely associated with the advancement of cell cycle, with a preference for the G0/G1 and S phases [23]. So, it can be speculated that cell cycle influences autophagic degradation, and the inhibition of autophagy may lead cells to be arrested to the G0/G1-phase.

The cell cycle is regulated by cyclins, cyclin-dependent kinases (CDKs) and CDK inhibitors, such as p21^Cip1 ^and p27^Kip1^associated proteins [24,25]. The distinct phases of the cell cycle are controlled by different cyclin/CDK complexes, which are activated in response to different extracellular signals. During G1 phase, the D-type cyclins (generally cyclin D1) are expressed in response to mitogenic signals, and by binding to CDK4 and CDK6, lead to activation of cyclin E/CDK2 and cyclin A/CDK2 in late G1 and S phase. Cyclin D1 regulates the physiologic cell proliferation by promoting progression through key checkpoints in late G1 phase of the cell cycle. As expected, the expression of cyclin D1 was down-regulated by the treatment of HT-29 cells with 5-FU for 24 to 48 h. However, it was not affected by the pre-treatment with CQ. Other essential regulators of the cell cycle are the cyclin-dependent kinase (CDK) inhibitors, such as p21^Cip1 ^and p27^Kip1^, which have inhibitory effects on several CDKs [26,27]. The p21^Cip1 ^and p27^Kip1 ^proteins specifically inhibit the complexes formed between CDK2 and cyclin E, which are required for entry into S phase from G1 phase [26,27]. Treatment of HT-29 cells with 5-FU resulted in decreased expressions of p21^Cip1 ^and p27^Kip1^, and simultaneously in increased expression of CDK2. The pre-treatment of cells with CQ inhibited the down-regulation of p21^Cip1 ^and p27^Kip1 ^expressions induced by 5-FU and, on the other hand, inhibited the up-regulation of the expression of CDK2. The expressions of cyclin E, CDK4 and CDK6 were not significantly affected by 5-FU and/or CQ treatments. Thus, we speculated that the resulting increased p21^Cip1 ^and p27^Kip1^, by binding to and consequently inhibiting the cyclin E/CDK2 complex activity, potentiated the cell cycle arrest in G0/G1 phase, as also reported by others [28]. Therefore, the up-regulation of p21^Cip1 ^and p27^Kip1 ^expressions in HT-29 cells induced by CQ, possibly contributed to the potentiation of the inhibitory effect of 5-FU, by promoting the cell cycle arrest to G0/G1 phase.

Accumulation of autophagosomes, which is observed by the treatment with CQ, could either indicate increased autophagic flux or defective autophagy. Recently, Wong CH et al. demonstrated the presence of a reactive oxygen species (ROS)-induced autophagy and apoptosis pathways in human tumor cell lines and in primary cells, in which H_2_O_2 _was implicated as the possible upstream stimulus[29]. They demonstrated an early increase in autophagic flux, but not defective autophagy, which contributed to cell death execution. Therefore, it was suggested that the non-classical pathways may be invariably associated with cell death, not working as a cell survival response[29]. The fate of the cells with accumulated autophagosomes, induced by CQ, is not definitely reported. However, in the colony-forming assay, we found that more than 90% of cells, treated with CQ alone for 12 h, were alive. Thus, it seems that the autophagosome accumulation induced by CQ has different implications than that observed in ROS-induced autophagy.

Recently, an area of growing interest is the potential contribution of these pro-survival events in promoting tumor cell resistance to chemotherapy. In this context, autophagy might play a role as a self-defense mechanism in 5-FU-treated colon cancer cells, and, thus, inhibition of autophagy may be an effective and attractive strategy to enhance the 5-FU-induced anti-cancer effect in colon cancer. Indeed, macroautophagy is remarkably up-regulated in colon cancer tissues when compared with the surrounding non-cancerous tissues, strongly supporting our hypothesis [30]. Since CQ is already clinically available, its use in clinical settings of anti-cancer therapy is feasible, compared with the other autophagy inhibitors, such as 3-MA, which need to have their safety to human beings confirmed prior to be tested in clinical trials.

However, it is notable that the long-term effects of prolonged use of a potent autophagy inhibitor may have unexpected side effects, as our understanding of the homeostatic role of autophagy in normal tissues is yet rudimentary [31]. Other combination therapies for 5-FU, such as Irinotecan, aspirin, proteasome inhibitor, 3-MA are being attempted [15,32-34], and CQ is a new candidate. In a previous study, we demonstrated that the pro-apoptotic effect of the chemopreventive agent, sulforaphane, on human colon cancer cells was potentiated by the pre-treatment of these cells with the specific inhibitor of autophagy, namely 3-MA [17].

## Conclusions

In summary, we clearly demonstrated that the combination therapy using CQ and 5-FU is an effective and promising strategy for the treatment of colorectal cancer. Since CQ is already in clinical use as the antimalarial drug, as well as an anti-inflammatory drug for the treatment of rheumatoid arthritis, lupus erythematosus and amoebic hepatitis, its application for the treatment of colorectal cancer may be feasible without need of phase I studies.

## Competing interests

The authors declare that they have no competing interests.

## Authors' contributions

KS, NHT, HN and KT actively participated in the design of the study. KS was the main researcher, performing most of the experiments, helped by NT, YS, KH, MK, and MH. The statistical analysis of the obtained resulted was conducted by KS, helped by KK and YO. NHT, JK, ES and GT revised the manuscript critically related to the adequateness of the methods, the significance of the results and the appropriateness of the interpretation of the results. HN gave the final approval of the version to be published. All authors read and approved the final manuscript for publication.

## Pre-publication history

The pre-publication history for this paper can be accessed here:

http://www.biomedcentral.com/1471-2407/10/370/prepub
